# MicroRNAs in atopic dermatitis: A systematic review

**DOI:** 10.1111/jcmm.15208

**Published:** 2020-04-30

**Authors:** Xin Yu, Meifang Wang, Linfeng Li, Lin Zhang, Matthew Tak Vai Chan, William Ka Kei Wu

**Affiliations:** ^1^ Department of Dermatology Beijing Friendship Hospital Capital Medical University Beijing China; ^2^ Department of Anaesthesia and Intensive Care and Peter Hung Pain Research Institute The Chinese University of Hong Kong Hong Kong Hong Kong; ^3^ State Key Laboratory of Digestive Diseases and LKS Institute of Health Sciences The Chinese University of Hong Kong Hong Kong Hong Kong

**Keywords:** atopic dermatitis, atopic eczema, inflammation, microRNAs

## Abstract

Atopic dermatitis (AD) is a chronic and recurrent inflammatory skin disease, affecting up to 10% to 20% of children and 3% of adults. Although allergen sensitization, skin barrier abnormalities and type 2 immune responses are involved, the exact molecular pathogenesis of AD remains unclear. MicroRNAs (miRNAs) are short (19‐25 nucleotides) single‐stranded RNA molecules that regulate gene expression at post‐transcriptional level and are implicated in the pathogenesis of many inflammatory and immunological skin disorders. This systematic review sought to summarize our current understanding regarding the role of miRNAs in AD development. We searched articles indexed in PubMed (MEDLINE) and Web of Science databases using Medical Subject Heading (MeSH) or Title/Abstract words (‘microRNA/miRNA’ and ‘atopic dermatitis/eczema’) from inception through January 2020. Observational studies revealed dysregulation of miRNAs, including miR‐143, miR‐146a, miR‐151a, miR‐155 and miR‐223, in AD patients. Experimental studies confirmed their functions in regulating keratinocyte proliferation/apoptosis, cytokine signalling and nuclear factor‐κB‐dependent inflammatory responses, together with T helper 17 and regulatory T cell activities. Altogether, this systematic review brings together contemporary findings on how deregulation of miRNAs contributes to AD.

## 1 INTRODUCTION

Atopic dermatitis (AD), or atopic eczema, is a common chronic and persistent inflammatory skin disease, affecting up to 10% to 20% of children and 3% of adults.^1^, ^2^ AD usually occurs in infancy but can also start or persist in adulthood, presenting a core challenge for dermatologists worldwide. The pathogenesis of AD remains largely unclear. Allergen sensitization, skin barrier abnormalities and type 2 immune responses (hallmarked by the differentiation of CD4^+^ T helper type 2 cells and the production of the type 2 cytokines, such as interleukins (IL)‐4, ‐5, ‐9 and ‐13) are considered to be key pathogenic processes contributing to AD development.^3^, ^4^ Patients with AD have also shown deregulated expression of T lymphocytes, as well as cytokines, antimicrobial peptides, chemokines, total immunoglobulin E (IgE), proteases and proteins critical for the normal structure of epithelial cells.^3^, ^4^

MicroRNAs (miRNAs) are short (19‐25 nucleotides) single‐stranded RNA molecules and were initially discovered in 1993.^5^ MiRNAs cannot be translated into proteins but can regulate expression of target genes post‐transcriptionally. Through base‐pairing between the seed region (nucleotide positions 2‐8) of miRNA and its target mRNAs, miRNA could guide the RNA‐induced silencing complex to their targets to induce their degradation and/or inhibit their translation.^6^ Altered expression of miRNAs has been documented in many kinds of diseases, including inflammatory and immunological skin disorders, which opened a novel area for researchers to understand pathogenesis, develop novel biomarkers and devise mechanism‐driven therapeutic strategies.^7^ Recent findings have demonstrated that miRNAs play a significant role in the pathogenesis of AD.

In this systematic review, we summarize current publications concerning the role of miRNAs in the development of AD. In addition, we discuss the potential use of miRNAs as diagnostic biomarkers and therapeutic targets in AD.

## 2 METHODS

### 2.1 Searching strategy and selection of studies

We searched articles indexed in PubMed (MEDLINE) and Web of Science databases using Medical Subject Heading (MeSH) or Title/Abstract words (‘microRNA/miRNA’ and ‘atopic dermatitis/eczema’) from inception through 9th January 2020. Although there was no initial limitation imposed on language during the search, only English‐based literature and non‐English studies with available English abstracts were further considered. We included any original study in which the role of miRNAs in AD was examined in relation to pathogenesis, diagnosis, prognosis and treatment with meeting abstracts and reviews excluded. The searching process was conducted independently by two investigators. Experts in the field of miRNAs or dermatology were involved in the literature analysis.

### 2.2 Ethical review

The present study is a systemic literature review. We do not involve human beings or experimental subjects in this study, and no any identifiable private information is collected.

## 3 RESULTS

**FIGURE 1:**
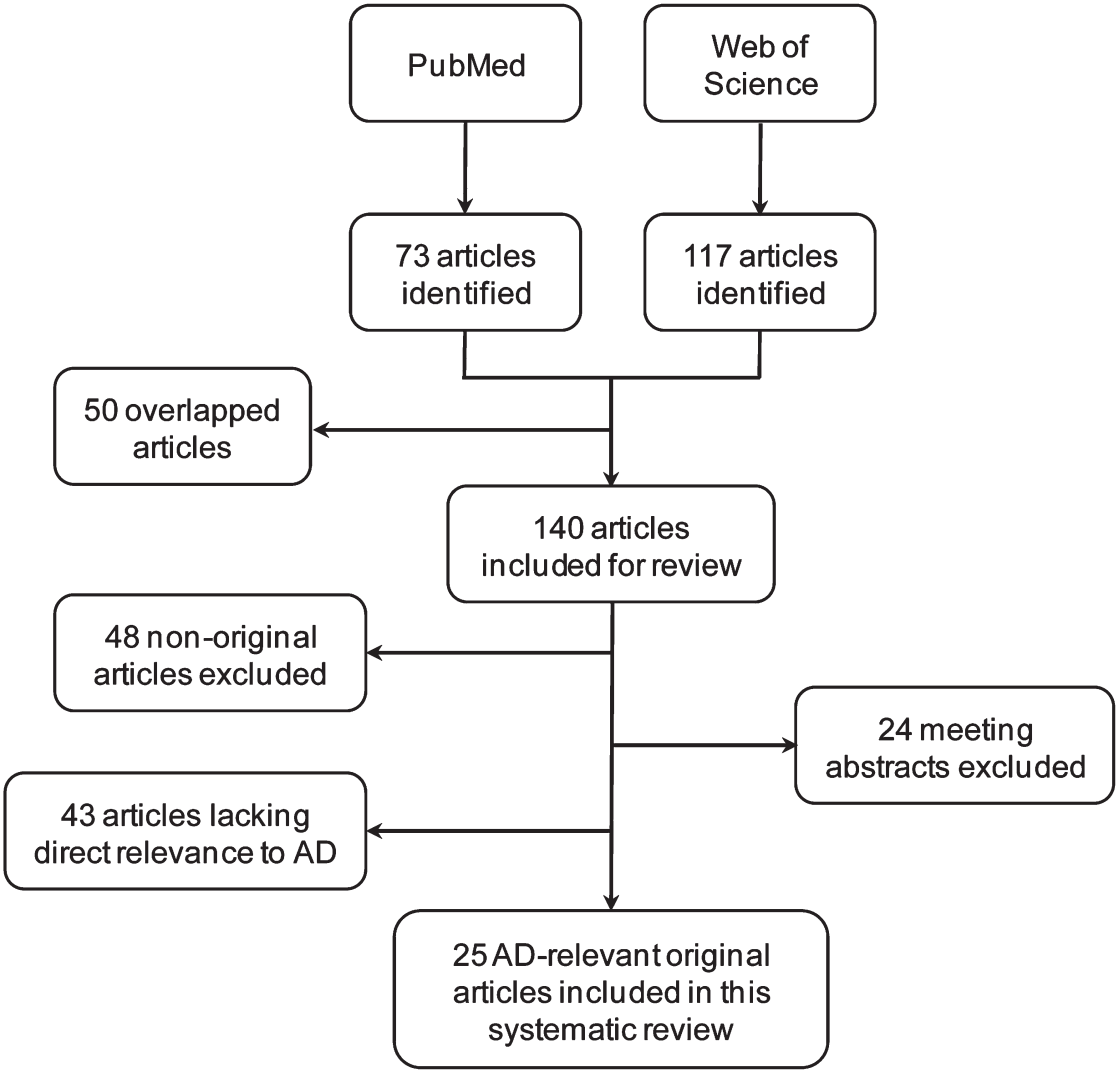
Flow chart of studies selection

A total of 73 items from PubMed and 117 items from Web of Science were found based on the search criteria, among which 25 original studies investigating miRNAs in AD were finally included in this systematic review. The papers excluded were either conference abstracts, not original articles, not directly related to AD, or lacking in evidence of dysregulation of the studied miRNA(s) in human AD patients (Figure 1).

### 3.1 MiRNA profiles in AD

Lv et al conducted genome‐wide miRNA profiling with serum and urine samples from AD patients.^8^ As compared with healthy children, serum levels of miR‐203 and miR‐483‐5p were significantly increased whereas urine miR‐203 level was markedly decreased in children with AD. Increased serum miR‐203 level was significantly associated with increased soluble tumour necrosis factor receptor I (sTNFRI) and sTNFRII, both of which are inflammatory markers. Moreover, reduced miR‐203 level in urine was significantly associated with abnormal serum IgE levels in AD patients.^8^ By RNA sequencing using plasma samples followed by validation with reverse transcription‐quantitative PCR, a recent study found that the circulating levels of miR‐194‐5p and miR‐184 were markedly reduced whereas let‐7d‐5p level was increased in children with AD.^9^ MiR‐146a and miR‐125b were also reported to show significantly higher and lower levels, respectively, in the serum of AD patients as compared with the control group.^10^, ^11^ Aside from normal blood samples, efforts have been made to profile miRNAs in umbilical cord serum from infants with subsequent diagnosis of AD. In this connection, increased miR‐144‐3p level in umbilical cord serum was associated with AD diagnosis at 1 year of age.^12^

MiRNA expression profiles in the skin lesions of AD patients have been determined by microarray.^13^, ^14^ In the study by Sonkoly et al, elevated expression of let‐7i, miR‐24, miR‐27a, miR‐29a, miR‐193a, miR‐199a and miR‐222 was reported.^13^ Gu et al also reported a multitude of dysregulated miRNAs (eg up‐regulation: miR‐4270, miR‐211, miR‐4529‐3p and miR‐29b; down‐regulation: miR‐184, miR‐135a and miR‐4454) in AD skin biopsies.^14^ Li et al performed bioinformatic analysis on miRNA microarray data deposited in the Gene Expression Omnibus database to identify differentially expressed miRNAs associated with AD.^15^ Three differentially expressed miRNAs, namely let‐7a, miR‐26a and miR‐143, were identified. Let‐7a was predicted to target ribonucleotide reductase regulatory subunit M2 (RRM2) and C‐C motif chemokine receptor 7 (CCR7), whereas miR‐26a might target hyaluronan synthase 3 (HAS3), DEP domain‐containing 1B (DEPDC1B), nicotinamide phosphoribosyltransferase (NAMPT), DENN domain‐containing 1B (DENND1B), a disintegrin and metalloproteinase domain 19 (ADAM19) and DEPDC1. miR‐143 potentially targets DENND1B.^15^ Aside from hybridization‐based microarray, Ichihara et al used PCR array to quantitate the expression of 88 miRNAs in three AD skin samples versus three normal skin samples, in which overexpression (fold‐change > 16) of miR‐520g, miR‐21, miR‐10b, miR‐223, and miR‐196a in AD lesions was demonstrated.^16^

MiRNAs are abundant in mammalian milk and may influence the risk of AD in infants. By small RNA sequencing, Simpson et al reported that differential abundance of several miRNAs, including miR‐146b‐5p, miR‐21‐5p, miR‐22‐3p, miR‐375 and let‐7f‐5p, in breast milk was associated with AD development by 2 years of age. Nevertheless, none of these miRNAs remained significant after correction for multiple testing.^17^

### 3.2 Overall significance of miRNAs in AD

Hener et al investigated the overall contribution of endogenous miRNAs as a whole in AD by assessing the effect of genetic ablation of Dicer (a double‐stranded RNA ribonuclease essential for miRNA maturation) in mouse epidermal keratinocytes in an experimental model of AD induced by the vitamin D3 analogue MC903.^18^ The investigators found that deletion of Dicer aggravated skin inflammation accompanied by an elevation of thymic stromal lymphopoietin (TSLP),^18^ whose overexpression per se was sufficient to initiate AD‐like inflammatory responses in mice.^19^ These data suggested that miRNAome in keratinocytes is in general anti‐inflammatory in AD pathogenesis.

### 3.3 Functional significance of specific miRNAs in AD

#### 3.3.1 MiR‐10a‐5p

Vaher et al reported that miR‐10a‐5p was up‐regulated in both non‐lesional and lesional skin of patients with AD as compared to healthy control skin.^20^ Transfection of miR‐10a‐5p into human primary keratinocytes reduced the number of cells in S‐phase and attenuated the induction of genes by IL‐1β related to cell cycle regulation, cell adhesion and cytokine signalling. HAS3, a damage‐associated positive regulator of keratinocyte proliferation and migration, was identified as the direct target of miR‐10a‐5p.^20^ These evidence collectively suggested that the aberrant up‐regulation of miR‐10a‐5p in AD could impair keratinocyte proliferation that is important for maintaining the skin barrier function.

#### 3.3.2 MiR‐29b

Gu et al demonstrated that miR‐29b was up‐regulated in lesional skin and sera from AD patients as compared with healthy individuals.^14^ Importantly, serum level of miR‐29b was correlated with the SCORAD value (a clinical score for assessing the extent and severity of AD). Functionally, miR‐29b mediated interferon (IFN)‐γ‐induced keratinocyte apoptosis by targeting Bcl‐2‐like protein 2 (BCL2L2),^14^ suggesting that aberrant up‐regulation of miR‐29b might contribute to AD‐associated epithelial barrier dysfunction.

#### 3.3.3 MiR‐124

Previous studies showed that miR‐124 was involved in inflammatory reaction. In addition, it was demonstrated to directly target nuclear factor (NF)‐κB in B‐cell lymphoma.^21^ Yang et al investigated the role of miR‐124 in AD and showed that miR‐124 expression was down‐regulated in chronic AD skin lesions.^22^ MiR‐124 expression could also be strongly inhibited by IFN‐γ and tumour necrosis factor (TNF)‐α. MiR‐124 inhibited p65 (a subunit of NF‐κB) expression, which played a crucial role in inflammation and immune response. Upon IFN‐γ or TNF‐α stimulation, IL‐8, chemokine (C‐C motif) ligand 5 (CCL5) and CCL8 expression were significantly down‐regulated by miR‐124 whereas they were up‐regulated by IFN‐γ or TNF‐α.^22^ Taken together, miR‐124 regulates inflammatory responses in keratinocytes and chronic skin inflammation in AD through regulating the NF‐κB pathway, indicating that restoring miR‐124 expression may be a potential therapeutic strategy for AD.

#### 3.3.4 MiRNA‐143

IL‐13, an important T helper 2 cytokine, has been demonstrated to impair normal epidermal barrier function. In this capacity, IL‐13 plays a critical role in many allergic diseases, such as asthma and AD.^23^ A study by Zeng et al showed that stimulation with IL‐13 suppressed miRNA‐143 expression in human epidermal keratinocytes.^24^ In addition, overexpression of miRNA‐143 in epidermal keratinocytes inhibited the luciferase activity of the vector containing 3′ untranslated region (UTR) of IL‐13 receptor alpha 1 (IL‐13Ra1) alongside repression of the IL‐13‐mediated down‐regulation of filaggrin, loricrin, and involucrin.^24^ Collectively, data suggested that miRNA‐143 could decrease IL‐13 activity and inflammatory reaction through targeting IL‐13Ra1 in epidermal keratinocytes. MiRNA‐143 may serve as a novel therapeutic target in AD patients.

#### 3.3.5 MiR‐146a

Previous studies showed that miR‐146a was an anti‐inflammatory miRNA with a compensatory up‐regulation in psoriasis.^13^ It was involved in TNF‐α signalling and the NF‐κB pathway.^25^ MiR‐146a expression was up‐regulated in keratinocytes and skin of AD patients.^26^ Transfection of miR‐146a decreased the expression of multiple pro‐inflammatory factors, including AD‐associated and IFN‐γ‐inducible genes CCL5, CCL8 and ubiquitin D (UBD) in keratinocytes and in a mouse model of AD. Inhibition of miR‐146a increased the expression of pro‐inflammatory factors in keratinocytes. Concordantly, miR‐146a‐deficient mice showed stronger inflammatory reaction, with increased accumulation of infiltrating cells in the dermis and elevated expression of inflammatory factors in the skin. MiR‐146a suppressed allergic skin inflammation partially through targeting the upstream mediators of NF‐κB signalling—IRAK1 and CARD10. In addition, human CCL5 was identified as a novel, direct target of miR‐146a.^26^ Aside from the NF‐κB pathway, a recent study reported the existence of a negative correlation between serum miR‐146a and IgE levels in patients with allergic‐type AD that is characterized by strong type‐2‐cell‐mediated immune response and remarkably high level of IgE in serum.^27^ Taken together, miR‐146a could limit NF‐κB‐dependent inflammatory reaction and type‐2‐cell‐mediated immune responses in AD. However, another study showed that miR‐146a could suppress keratinocyte proliferation.^28^ Whether miR‐146a up‐regulation could contribute to epithelial barrier dysfunction in AD remains unclear.

#### 3.3.6 MiR‐151a

MiR‐151a belongs to the miR‐28 family. Its host gene, focal adhesion kinase (FAK), is located on chromosome 8q. Chen et al showed that miR‐151a was involved in the pathogenesis of AD by regulating IL‐12 receptor β2 (IL12RB2), a subunit of IL‐12 receptor.^29^ MiR‐151a level was significantly higher in the plasma of AD patients as compared with the healthy individuals. Functionally, overexpressing miR‐151a in human T helper cells significantly down‐regulated IL12RB2 expression.^29^

#### 3.3.7 MiR‐155

MiR‐155 is implicated in the regulation of innate and adaptive immune responses. In particular, miR‐155 is necessary for the differentiation of T helper type 17 (Th17) cells in autoimmune diseases.^30^, ^31^ Sonkoly et al demonstrated that miR‐155 was one of the most up‐regulated miRNAs in AD patients.^32^ MiR‐155 expression was predominantly found in infiltrating immune cells and was up‐regulated during T cell activation. Moreover, miR‐155 expression was induced by T cell activators in peripheral blood mononuclear cells and allergens in the skin. Cytotoxic T lymphocyte antigen 4 (CTLA‐4), a critical negative regulator of T cell activation, was determined as the direct target of miR‐155. Overexpression of miR‐155 decreased CTLA‐4 levels and increased proliferation in T helper cells,^32^ indicating that aberrant up‐regulation of miR‐155 could promote chronic skin inflammation by increasing T helper cell proliferation through targeting CTLA‐4. Ma et al also demonstrated that miR‐155 was overexpressed in AD patients and positively correlated AD severity. In addition, percentage of Th17 cells was increased in AD patients and there was a positive correlation between miR‐155 expression and Th17 cell percentage.^33^ However, in another study involving 23 AD patients and 23 healthy individuals, a significant correlation between miR‐155 and the Th17‐to‐regulatory T cell (Treg) ratio could not be demonstrated.^34^ In a murine model of AD recurrence, an integrative analysis of miRNAs, long non‐coding RNAs and mRNAs suggested the central role of miR‐155 in the regulation of protein kinase inhibitor α (PKIα) through competition with lncRNA0490+.^35^ Functionally, silencing of miR‐155 alleviated AD‐associated epidermal thickening and reduced inflammatory cell infiltration via up‐regulating PKIα and thus enhancing epithelial tight junction formation.^36^ These findings demonstrated that miR‐155 is involved in AD pathogenesis by regulating both cytokine responses and epithelial barrier function. Nevertheless, more research is needed to confirm the involvement of Th17 cells.

Genetic variations in *miR‐155* gene might contribute to AD susceptibility. Sääf et al reported that the expression of *BIC* gene which encodes the precursor of miR‐155 was increased in AD skin as compared with healthy controls.^37^ Importantly, 3 out of 5 single nucleotide polymorphisms (SNPs) covering the *BIC/miR‐155* gene were found to be associated with AD (*P* < .05). Nevertheless, such associations were not statistically significant after correction for multiple testing.^37^

#### 3.3.8 MiR‐223

MiR‐223 expression is predominantly found in neutrophils, monocytes and eosinophils and is associated with tobacco smoking.^38^, ^39^ Herberth et al investigated the associations among prenatal tobacco smoke exposure, miRNAs and Treg cell number.^39^ Maternal and cord blood miR‐223 expression levels were positively correlated with maternal urine cotinine level. Maternal miR‐223 expression was also associated with indoor concentrations of benzene and toluene. In addition, increased maternal and cord blood miR‐223 expression was correlated with lower Treg cell number, in which lower Treg cell number at birth has been shown to increase the risk of AD in children during the first 3 years of life.^39^ Concordantly, a recent study demonstrated the significant elevation of miR‐223 level in the whole blood of AD patients.^40^ Altogether, prenatal maternal tobacco smoke might increase blood miRNA‐223 level, which in turn regulates children's cord blood Treg cell number and AD risk.

## 4 CONCLUSIONS AND DISCUSSION

**FIGURE 2:**
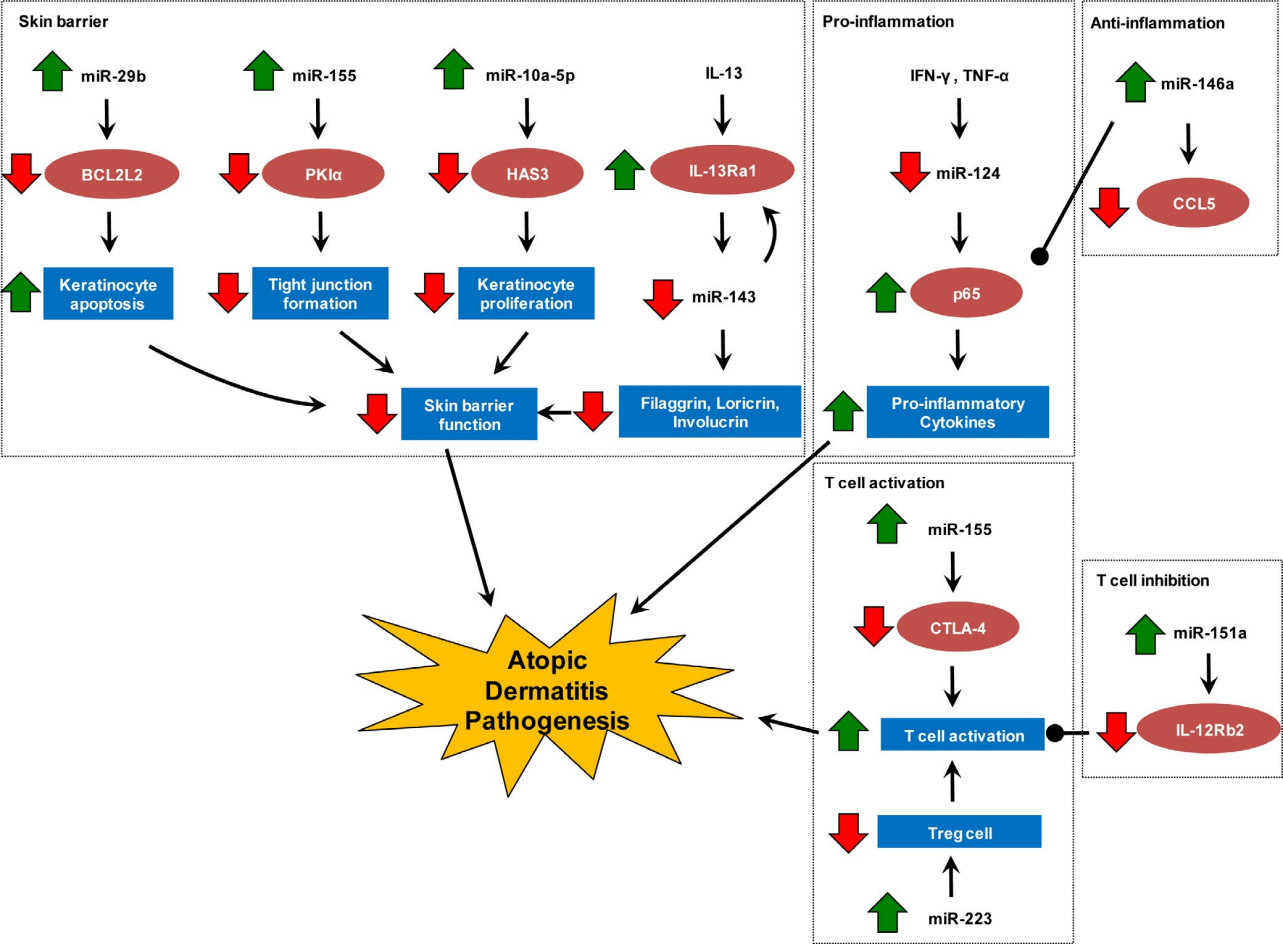
Functions of deregulated miRNAs in the pathogenesis of atopic dermatitis

Through profiling and functional studies, the roles of miRNAs in AD are just emerging. Mechanistic investigations have linked miRNA dysregulation to aberrant skin barrier function, cytokine signalling and NF‐κB‐dependent inflammatory responses, together with Th17 and Treg activities (Table 1 and Figure 2). Nevertheless, the functions of many AD‐associated miRNAs remain obscured. Further studies are needed to systemically assess the involvement of these miRNAs in AD pathogenesis.

**TABLE 1:** The functions of miRNAs in atopic dermatitis

miRNAs	Mechanism of action	Target cells	Target mRNA
miR‐10a‐5p	Inhibition of keratinocyte proliferation	Epidermal keratinocytes	HAS3
miR‐29b	Promotion of INF‐γ‐induced keratinocyte apoptosis	Epidermal keratinocytes	BCL2L2
miR‐124	Inhibition of inflammatory responses	Epidermal keratinocytes	RELA (p65 subunit of NF‐κB)
miR‐143	Suppression of IL‐13‐induced dysregulation of skin barrier proteins	Epidermal keratinocytes	IL‐13Rα1
miR‐146a	Suppressing the expression of many pro‐inflammatory factors	Epidermal keratinocytes	IRAK1, CARD10, CCL5
miR‐151a	Inhibition of IL‐12 signalling	T helper cells	IL12RB2
miR‐155	Promotion of Th17 differentiation	T cells	CTLA‐4
	Inhibition of tight junction formation	Epidermal keratinocytes	PKIα
miR‐223	Positive correlation with Treg cell number	Not specified	Not specified

The above‐mentioned biological processes regulated by miRNAs have important therapeutic implications. For instances, barrier‐restoring therapies have aroused interest in AD treatment research.^41^ A NF‐κB inhibitor has also been shown to alleviate disease severity in a mouse AD model.^42^ Moreover, agents antagonizing Th17 showed favourable outcomes in recent AD clinical trials.^43^ It has also been promulgated that therapeutic Treg amplification might suppress the allergic inflammatory cascade in AD.^44^ These emerging themes represent future directions of new AD drug development. To this end, experimental validation of AD‐associated miRNAs and their downstream mediators as druggable targets will undoubtedly facilitate the development of mechanism‐driven therapy for this refractory dermatological condition of which treatment options are currently very limited. Research effort should also be put forth to optimize the way to deliver miRNA mimics or inhibitors in a cell‐type‐specific manner as the same miRNA could have divergent actions in different tissues.

The literature has ample examples illustrating the use of miRNAs as novel diagnostic biomarkers.^45^ However, the results of miRNA studies in AD are very often not conclusive as the samples used for analysis were relatively small in number and heterogeneous. Further investigations into miRNA‐based diagnostic biomarkers should involve larger cohorts of AD patients in a multi‐centre setting. With more translational research, it is hopeful that miRNA‐based diagnostics and therapeutics will become part of our clinical practice for AD management in the near future.

## CONFLICT OF INTEREST

The authors declare no competing financial interests.

## AUTHOR CONTRIBUTIONS

Xin Yu, Meifang Wang, Linfeng Li, Matthew TV Chan and William KK Wu contributed to research conception, designed the study and wrote the manuscript.

## Data Availability

The authors confirm that they have included a citation for available data in their references section.
